# Derivation of a blood count-based machine learning model to prioritise sequencing for high-risk clonal haematopoiesis: a retrospective cohort study

**DOI:** 10.1016/j.eclinm.2026.104238

**Published:** 2026-09-22

**Authors:** Riya Nandi, Kun Zhao, Yash Pershad, Nabi Nazmoon Khondoker, Caitlyn Vlasschaert, Md Mesbah Uddin, Pradeep Natarajan, Daniel J. DeAngelo, Marlise R. Luskin, R. Coleman Lindsley, Hajime Uno, Shai Shimony, Alexander G. Bick, Lachelle D. Weeks

**Affiliations:** a Division of Population Sciences, Department of Medical Oncology, Dana-Farber Cancer Institute, Boston, MA, USA; b Division of Genetic Medicine, Department of Medicine, Vanderbilt University Medical Center, Nashville, TN, USA; c Harvard T.H. Chan School of Public Health, Boston, MA, USA; d Queens University, Kingston, Ontario, Canada; e Heart and Vascular Institute, Massachusetts General Brigham, Boston, MA, USA; f Program in Medical and Population Genetics, Broad Institute of Harvard and MIT, Cambridge, MA, USA; g Department of Medicine, Harvard Medical School, Boston, MA, USA; h Division of Hematologic Malignancies, Department of Medical Oncology, Dana-Farber Cancer Institute, Boston, MA, USA

**Keywords:** Clonal haematopoiesis, High-risk clonal haematopoiesis, Pre-sequencing risk model, PRISM, Blood counts, Myeloid neoplasia

## Abstract

**Background:**

Clonal haematopoiesis is a precursor of myeloid neoplasia and common in older adults, Precursor risk stratification is critical to early cancer detection, guiding surveillance and prevention efforts to high-risk populations. Existing clonal haematopoiesis risk scores use molecular features to estimate risk of myeloid neoplasia, but only around 1% of adults with detectable clonal haematopoiesis are at high risk of myeloid neoplasia transformation. Moreover, sequencing based molecular diagnostics are expensive with limited global availability highlighting the need for pre-sequencing strategies which identify populations enriched for individuals with high-risk clonal haematopoiesis.

**Methods:**

We used blood count parameters, age, and sex as input features to and develop a multi-class machine learning model that pre-classifies individuals based on their probability of having high-risk clonal haematopoiesis or developing future myeloid neoplasia. Stratified splitting was used to divide 461,576 eligible UK Biobank participants (recruited October 1, 2006 to March 13, 2010) into a training cohort for model derivation (n = 369,260) and a test cohort for internal validation (n = 92,316). Ground truth output class assignments were defined based on documented progression to incident myeloid neoplasia and presence of clonal haematopoiesis with high-risk features defined using molecular parameters of the clonal haematopoiesis risk score. Individuals were labelled positive if they had high-risk clonal haematopoiesis and progressed to myeloid neoplasia, negative if they lacked clonal haematopoiesis with high-risk features, and equivocal if they had higher risk features but had no documented incident myeloid neoplasia event. We then compared model predictions against ground truth labels for validation in the UKB test cohort as well as in independent clinical and population cohorts from Dana-Farber Cancer Institute Hematologic Malignancy Data Repository (HMDR; n = 2233; March 26, 2010, to June 5, 2024) and the All of US database (n = 14,674; May 31, 2017 to October 1, 2023), respectively.

**Findings:**

The resulting pre-sequencing risk model (PRISM) categorises all cases of high-risk clonal haematopoiesis as positive and pre-classifies 40% (2158 of 5383) of low-risk clonal haematopoiesis and 55% (47,894 of 86,429) of clonal haematopoiesis-negative cases as negative. Validation of PRISM revealed an area under the curve of 0.89 (95% CI 0.85–0.93) in the UK Biobank test cohort, 0.74 (0.61–0.84) in HMDR, and 0.87 (0.77–0.96) in All of US. Negative predictive values for negative class assignments were 0.993 (95% CI 0.992–0.993) in the UKB test cohort, 0.968 (0.943–0.981) in HMDR, and 0.998 (0.997–0.999) in All of US.

**Interpretation:**

PRISM positive class assignment defines a population most enriched for high-risk clonal haematopoiesis, while negative class predictions, mark individuals without high-risk clonal haematopoiesis. PRISM therefore provides a framework for reproducibly assigning blood count-based risk thresholds to prioritise clonal haematopoiesis screening in populations at risk for myeloid neoplasia.

**Funding:**

Damon Runyon Cancer Research Foundation, Edward P Evans Foundation for MDS, and the National Heart Lung and Blood Institute.

**Research in context**

**Evidence before this study**

Clonal haematopoiesis is a recognised precursor of blood cancers that is also associated with other adverse health effects, including cardiovascular diseases. Clinically used tools for risk stratification, including the clonal haematopoiesis risk score reveal that a high-risk minority, around 1% of adults with clonal haematopoiesis, have a clinically significant risk of transformation to blood cancers including acute myeloid leukaemia or myelodysplastic syndrome. We used the terms “high-risk clonal haematopoiesis”, “clonal haematopoiesis,” “prediction of myeloid neoplasia” and “blood cancer risk prediction” to search PubMed (database inception to Dec 31, 2025) for human studies related to prediction of high-risk clonal haematopoiesis. We found no publications focused on predicting high-risk clonal haematopoiesis as defined by clinical risk algorithms like the clonal haematopoiesis risk score. However, one study derived models which successfully predicted specific clonal haematopoiesis genotypes. Although regression-based models using electronic health data inputs have been developed to discriminate pre-acute myeloid leukaemia cases from low-risk clonal haematopoiesis cases there are no validated models that guide which populations should even be tested for clonal haematopoiesis. Strategies that identify populations potentially enriched for high-risk clonal haematopoiesis would result in more efficient use of molecular diagnostic testing for clonal haematopoiesis and limit overdiagnosis in low-risk populations with minimal myeloid neoplasia risk.

**Added value of this study**

This study derives and robustly validates a machine learning-based pre-sequencing risk model (PRISM), that uses complete blood count parameters to classify individuals based on their likelihood of having high-risk clonal haematopoiesis and guide the use of molecular profiling. Using PRISM, all individuals with high-risk clonal haematopoiesis are classified as positive. PRISM negative class labels adults who do not have high-risk clonal haematopoiesis and are unlikely to benefit from testing (negative predictive value > 0.96).

**Implications of all the available evidence**

PRISM is the first validated pre-sequencing model for clonal haematopoiesis. By using routinely available complete blood count data as inputs, PRISM provides a reproducible framework for identifying populations enriched for high-risk clonal haematopoiesis. In so doing, PRISM allows pre-sequencing estimation of the potential value of molecular profiling for clonal haematopoiesis in adults and offers a strategy to limit overdiagnosis of age-related clonal haematopoiesis of limited clinical consequence. With further optimisation and prospective validation, PRISM may emerge as decision making tools for patients, clinicians, and consumers considering the value of clinical, research-based, or emerging direct-to-consumer testing for clonal haematopoiesis.

## Introduction

Population studies have identified expanded clones of haematopoietic cells possessing mutations in genetic drivers of blood cancers in individuals without any evidence of malignancy.^1^, ^2^, ^3^, ^4^ This state, known as clonal haematopoiesis, is associated with age and multiple malignant and non-malignant adverse health outcomes.^2^ Two subtypes, clonal haematopoiesis of indeterminate potential (CHIP) and clonal cytopenia of undetermined significance (CCUS), have been formally defined due to their clinical relevance as precursors of myeloid neoplasm.^5^^,^^6^ Hereafter, we use clonal haematopoiesis to refer exclusively to CHIP or CCUS, without distinction. Clonal haematopoiesis is detected in up to 20% adults aged 65 years older; however, transformation to myeloid neoplasia occurs at a rate of only 0.5–1.0% per year.^7^ While most individuals with clonal haematopoiesis will not progress to overt myeloid neoplasia, the risk of progression is highly heterogeneous, being as high as 90% in some groups.^8^

Several molecular and clinical features predict the risk of malignant evolution of clonal haematopoiesis.^8^, ^9^, ^10^, ^11^, ^12^, ^13^, ^14^ Prognostic features have been combined to create the clonal haematopoiesis risk score (CHRS)^9^ and similarly useful tools^10^^,^^11^ to estimate the risk of transformation from clonal haematopoiesis to myeloid neoplasia. The CHRS integrates molecular predictors (presence of a single *DNMT3A* mutation, presence of high-risk mutations, variant allele fraction of 20% or more, and presence of more than one mutation), laboratory features (red cell distribution width [RDW], mean corpuscular volume [MCV], and presence of cytopenias), and age to classify individuals as high-, intermediate-, or low-risk for progression to myeloid neoplasia.^9^ Despite its utility, only ∼1% of adults with clonal haematopoiesis detected in population-scale sequencing will be high-risk by CHRS (10-year myeloid neoplasia incidence of 52%) and around 90% of clonal haematopoiesis cases will be low risk (10-year myeloid neoplasia incidence <1%).^9^ In contrast with population screening for other pre-malignant lesions, large-scale deployment of molecular diagnostics for clonal haematopoiesis is impractical owing to the large proportion of low-risk cases and global limitations for genomic testing infrastructure in oncology screening systems.^15^ Thus, strategies that guide the use of clonal haematopoiesis diagnostics towards populations enriched for high-risk clonal haematopoiesis would mitigate risks associated with overdiagnosis of low-risk clonal haematopoiesis^16^^,^^17^ while aiding clinical and research decision making.

We hypothesised that molecular features of clonal haematopoiesis are reflected in blood cell counts and morphologies, enabling the use of complete blood cell count (CBC) data inputs in machine learning models to predict the presence of high-risk clonal haematopoiesis. We leveraged population and clinical databases to develop a tree-based machine learning pre-sequencing risk model (PRISM) that groups populations into three distinct classes: a positive class requiring a follow-up sequencing to diagnose high-risk clonal haematopoiesis; a negative class for whom sequencing may be deferred due to low likelihood of clinically significant clonal haematopoiesis; and an equivocal class for whom CBC information alone is insufficient for clinical decision making. PRISM class assignments establish a pre-sequencing risk threshold useful for directing screen for clonal haematopoiesis with sequencing assays towards populations enriched for high-risk clonal haematopoiesis and myeloid neoplasia risk.

## Methods

### Data sources

UK Biobank (UKB) data were extracted under application 50,834 from a cohort of 502,490 participants aged 40–69 years recruited between October 1, 2006 and March 13, 2010. Somatic variant detection from whole-exome sequencing was previously described^9^^,^^18^ and we used the presence of somatic variants in at least one gene associated with clonal haematopoiesis or myeloid neoplasia at variant allele fraction (VAF) of 2% or greater to define individuals as clonal haematopoiesis-positive. Samples for sequencing and lab values were drawn on the same day. We excluded participants with previous haematological malignancy diagnoses as well as those for whom myeloid neoplasia was not the incident haematological malignancy. Bone marrow evaluation for myeloid neoplasia was not performed for participants at baseline. After removing participants with unavailable whole-exome sequencing or missing complete blood count (CBC) data, 461,576 participants were eligible for study inclusion (Supplementary Fig. S1). To ensure uniform output class distributions, we used stratified splitting to divide eligible participants into a training cohort (n = 369,260 [80%]) and an unseen test cohort (n = 92,316 [20%]). The training cohort was used to train the multiclass prediction model while the unseen test cohort from UKB and the two additional external cohorts described below were used for model validation.

The Dana-Farber Hematologic Malignancies Data Repository (HMDR) registry contained 4132 individuals without haematological malignancy diagnosis evaluated in haematology clinics at Dana-Farber between March 26, 2010, and June 5, 2024. After eliminating individuals with missing clinical sequencing or who lacked CBC data obtained within a month of sequencing, an eligible cohort of 2233 clinical cases remained for external validation (Supplementary Fig. S2). Clonal haematopoiesis status was assigned to eligible participants based on the presence of defining somatic variants at VAF of 2% or greater in sequencing using a targeted clinical haematology panel (at around 40 × depth) and the presence or absence of cytopenias per WHO 5th edition criteria.^5^ The HMDR clinical cohort had variable amounts of missingness for CBC parameters. At least one CBC parameter was missing from 1651 (74%) of 2233 of the HMDR cohort, with 1246 (55%) missing reticulocyte counts, 610 (27%) missing eosinophil counts, 604 (27%) missing lymphocyte counts, 604 (27%) missing monocyte counts, 604 (27%) missing neutrophil counts, 164 (7%) missing platelet crit measurements, 13 (<1%) missing MCV, 13 (<1%) missing RBC, 13 (<1%) missing RDW, 13 (<1%) missing haemoglobin, and 13 (<1%) missing platelet counts. Multi-parameter missingness was as follows: 25 (1%) were missing five or more, 350 (16%) four, 1 (<1%) three, and 92 (4%) two CBC parameters. Missing variables were imputed using k-nearest neighbours imputation.

The All of Us Research Program population database is a USA-based population cohort with integrated electronic health record, survey, and whole genome sequencing data from individuals consented to study participation between May 31, 2017 and October 1, 2023. Clonal haematopoiesis variant calls and laboratory data were available for 276,542 individuals in the All of US database. After retaining only participants whose sequencing data were obtained within 30 days of CBC data collection, 14,674 individuals were eligible for inclusion in a second external validation cohort (Supplementary Fig. S3). Clonal haematopoiesis calling in All of US was performed using available whole genome sequencing data (mean depth around 40 × ). Clonal haematopoiesis-defining somatic single nucleotide variants and short insertions/deletions (indels) with at least 2% VAF in 74 genes associated with clonal haematopoiesis were identified using established Mutect2-based variant detection pipelines.^19^ As in UKB, bone marrow evaluation for myeloid neoplasia was not systematically performed. The final All of US cohort (n = 14,674) lacked giant platelet and platelet crit values. Missingness was addressed using k-nearest neighbours imputation of values in the unselected cohort (n = 276,542).

### Model derivation

To define model output classes, we first assigned a molecular clonal haematopoiesis (m-CH) score to everyone which represented risk of progression to myeloid neoplasia. Myeloid neoplasia outcomes were defined using ICD-10 codes (Supplementary Table S1). The m-CH score values ranged from 0 to 7.5 and were derived from the sum of scores assigned to clonal haematopoiesis risk score (CHRS) molecular predictors^9^: presence of high-risk genes (*JAK2*, *SRSF2*, *SF3B1*, *ZRSR2*, *TP53*, *RUNX1*, *IDH1*, *IDH2*, and *FLT3*), having one mutation in *DNMT3A* as the sole molecular abnormality (single *DNMT3A*), having two or more mutations, and maximum VAF of 20% or more (Supplementary Table S2). We then selected an m-CH score threshold above which myeloid neoplasia was most likely to occur, to distinguish myeloid neoplasia-predisposed individuals from individuals unlikely to develop myeloid neoplasia. Ground truth outcome classes were then assigned as positive (m-CH above threshold and incident myeloid neoplasia), equivocal (m-CH above threshold and no incident myeloid neoplasia), and negative (m-CH below the threshold). Limitations in sensitivity of detection for clonal haematopoiesis variants could influence clonal haematopoiesis–genotype associations that define the m-CH score.^20^ However, because VAFs trend larger for high-risk clonal haematopoiesis, this has minimal impact on output class assignments.

We considered 23 available blood count parameters, age, and sex as putative input features (Supplementary Fig. S4A). Then, we used Spearman’s correlation analysis (Supplementary Fig. S4B) to identify highly correlated features (correlation coefficient ≥0.9), resulting in the elimination of white blood cell count, haematocrit, and high-scattering reticulocyte count from the input feature list to reduce multicollinearity. Given the absence of platelet distribution width for the external validation sets, we used platelet distribution width to define a threshold for giant platelets in UKB data (Supplementary Fig. S5) and substituted “giant platelets” as a model input feature.

A balanced version of the UKB training set was generated dynamically within each training fold using an imbalanced-learn pipeline that applied the Synthetic Minority Over-sampling technique augment the minority (positive) class, followed by random under sampling of the majority classes. A final resampled training set containing 3000 individuals, 1000 per class, was used to select model architecture. We used the remaining 20 input features to compare area under the receiver operating characteristic curves (AUCs) of nine classifiers trained on the resampled subset of the UKB training set and selected Balanced Random Forest^21^^,^^22^ as the best performing model architecture (Supplementary Fig. S4C). After tuning hyperparameters using random search and maximising macro-averaged recall across 10-fold cross validation, we used the Boruta^23^ algorithm to eliminate sex, mean corpuscular haemoglobin concentration, basophil count, mean platelet volume, and nucleated red blood cell count as features that were non-essential to the model’s decision-making process (Supplementary Fig. S4D). Finally, we eliminated reticulocyte volume, mean sphere cell volume, mean corpuscular haemoglobin, and immature reticulocyte count, as features that were inconsistently annotated in CBC data in external validation datasets. Optimal thresholds based on Youden’s criteria were set for each class while making predictions. Model performance parameters were insignificantly altered before or after feature elimination (Supplementary Table S3).

### Cardiovascular disease outcomes

Incident cardiovascular disease outcomes were defined as events occurring after clonal haematopoiesis diagnosis using ICD-10 codes for myocardial infraction, angina, atherosclerosis, ischaemic heart failure, ischaemic stroke and other ischaemic cardiovascular diseases (Supplementary Table S1).

### Ethics

De-identified research data were accessed via application from UKB (application #50834) and All of US (curated data repository version 8, controlled tier C2024Q3R4). Dana-Farber Institutional Review Board (IRB) pre-deemed planned analyses of these datasets as non-human subjects research due to the use of de-identified data. Analysis of HMDR data was approved by the Dana Farber IRB under DFCI protocol #24-373, which granted a waiver of informed consent.

### Statistics

All computations were performed in Python version 3.9.6. Kaplan–Meier survival curves were estimated using the lifelines package in Python with differences between groups assessed using the two-sided log-rank tests and confidence intervals determined by the log–log transformed Greenwood method. Hazard ratios were determined using multivariate Cox proportional hazard models. Balanced Random Forest classifier from Python’s imbalanced-learn module was used for PRISM derivation. For model performance metrics, 95% CIs were calculated using the Wilson confidence interval given small sample sizes. For all statistical tests, significance was defined as a p value of less than 0.05.

### Role of funding source

Funders were not involved in study design, manuscript writing, data collection, analysis, or interpretation.

## Results

Baseline cohort characteristics are included in Table 1. The UKB cohort was split into a training set (n = 369,260 [80%]) and a withheld test set (n = 92,316 [20%]). Within the training set, median follow-up was 13 years (IQR 12.3–13.6), and incident myeloid neoplasia events 1317 participants (0.36%). Clonal haematopoiesis was detected in 22,990, including 1743 with CCUS; no clonal haematopoiesis mutation was detected in 346,270. Mutational profiles for each dataset are shown in Supplementary Fig. S6 and are consistent with previous reports.^9^^,^^11^

**Table 1:** Baseline cohort characteristics.

Variable	UK Biobank training set (n = 369,260)	UK Biobank test set (n = 92,316)	HMDR (n = 2233)	All of us (n = 14,674)
Mean age, years (SD)	57.03 (8.09)	57.01 (8.07)	63.29 (16.25)	54.95 (16.74)
Sex				
Male	169,305 (45.8%)	42,314 (45.8%)	989 (44.3%)	5643 (38.5%)
Female	199,955 (54.2%)	50,002 (54.2%)	1244 (55.7%)	9031 (61.5%)
Follow-up time, median (range)	12.9 years (0–15)	12.9 years (0–15)	4.9 years (0–9.9)	2.1 years (0.5–4)
Cytopenia				
Yes	26,418 (7.2%)	6633 (7.2%)	1420 (63.6%)	3332 (22.7%)
No	342,842 (92.8%)	85,683 (92.8%)	813 (36.4%)	11,342 (77.3%)
Clonal haematopoiesis status				
Clonal hematopoiesis of indeterminate potential	21,247 (5.8%)	5429 (5.9%)	206 (9.2%)	533 (3.6%)
Clonal cytopenia of undetermined significance	1743 (0.47%)	458 (0.5%)	602 (27%)	183 (1.25%)
No clonal haematopoiesis	346,270 (93.8%)	86,429 (93.6%)	1425 (63.8%)	13,958 (95.1%)
Myeloid neoplasia outcomes				
Incident myeloid neoplasia	1317 (0.36%)	335 (0.36)	20 (0.9%)	154 (1.05%)
No Incident myeloid neoplasia	367,943 (99.64%)	91,981 (99.64)	2213 (99.1%)	14,520 (98.95%)
CHRS^a^				
Low	21,009 (5.7%)	5383 (5.83%)	259 (11.6%)	516 (3.5%)
Intermediate	1831 (0.5%)	462 (0.5%)	373 (16.7%)	183 (1.3%)
High	150 (0.04%)	42 (0.04%)	176 (7.9%)	17 (0.11%)
Molecular clonal haematopoiesis score				
≤4	364,090 (98.6%)	91,024 (98.6%)	1798 (80.5%)	14,475 (98.6%)
>4	5170 (1.4%)	1292 (1.4%)	435 (19.5%)	199 (1.4%)
Outcomes^b^				
m-CH score ≤ 4	364,090 (98.6%)	91,024 (98.6%)	1798 (80.5%)	14,475 (98.6%)
m-CH score > 4 without incident myeloid neoplasia	4876 (1.3%)	1219 (1.32%)	417 (18.7%)	189 (1.3%)
m-CH score > 4, with incident myeloid neoplasia	294 (0.08%)	73 (0.08%)	18 (0.8%)	10 (0.07%)

Data are n (%) unless otherwise specified. A training cohort from the UK Biobank was used to train the balanced random forest model and the test set was used for internal validation. External validation was performed on the HMDR and All of Us cohorts.

CHRS = clonal haematopoiesis risk score. HMDR = Dana-Farber Cancer Institute Hematologic Malignancy Data Repository. m-CH = molecular clonal hematopoiesis.

a CHRS is determined as indicated in ref.^9^

b m-CH is derived from the sum of CHRS molecular features.

Molecular risk was established using m-CH scores (Fig. 1), with clonal haematopoiesis-negative individuals assigned an m-CH score of 0. The distribution of m-CH scores ranged from 3.5 to 7.5 for individuals with clonal haematopoiesis (Fig. 1A). An myeloid neoplasia risk threshold was established by comparing m-CH scores between the UKB training set subpopulations with and without myeloid neoplasia. Nearly all individuals who developed incident myeloid neoplasia (Fig. 1B and C) or were classified as high- or intermediate-risk by the CHRS^9^ (Fig. 1D) were captured by an m-CH score of more than 4, defining this as the m-CH threshold for myeloid neoplasia predisposition. Ground truth output for each participant was subsequently labelled (Fig. 1E) as negative for 364,090 (98.6%) individuals (m-CH score ≤4), positive for 294 (0.08%) individuals (m-CH score >4 with incident myeloid neoplasia diagnosis), and equivocal for 4876 (1.3%) individuals (m-CH score >4 without incident myeloid neoplasia diagnosis). Ground truth labels were similarly applied to internal and external validation cohorts (Table 1).

**Fig. 1:**
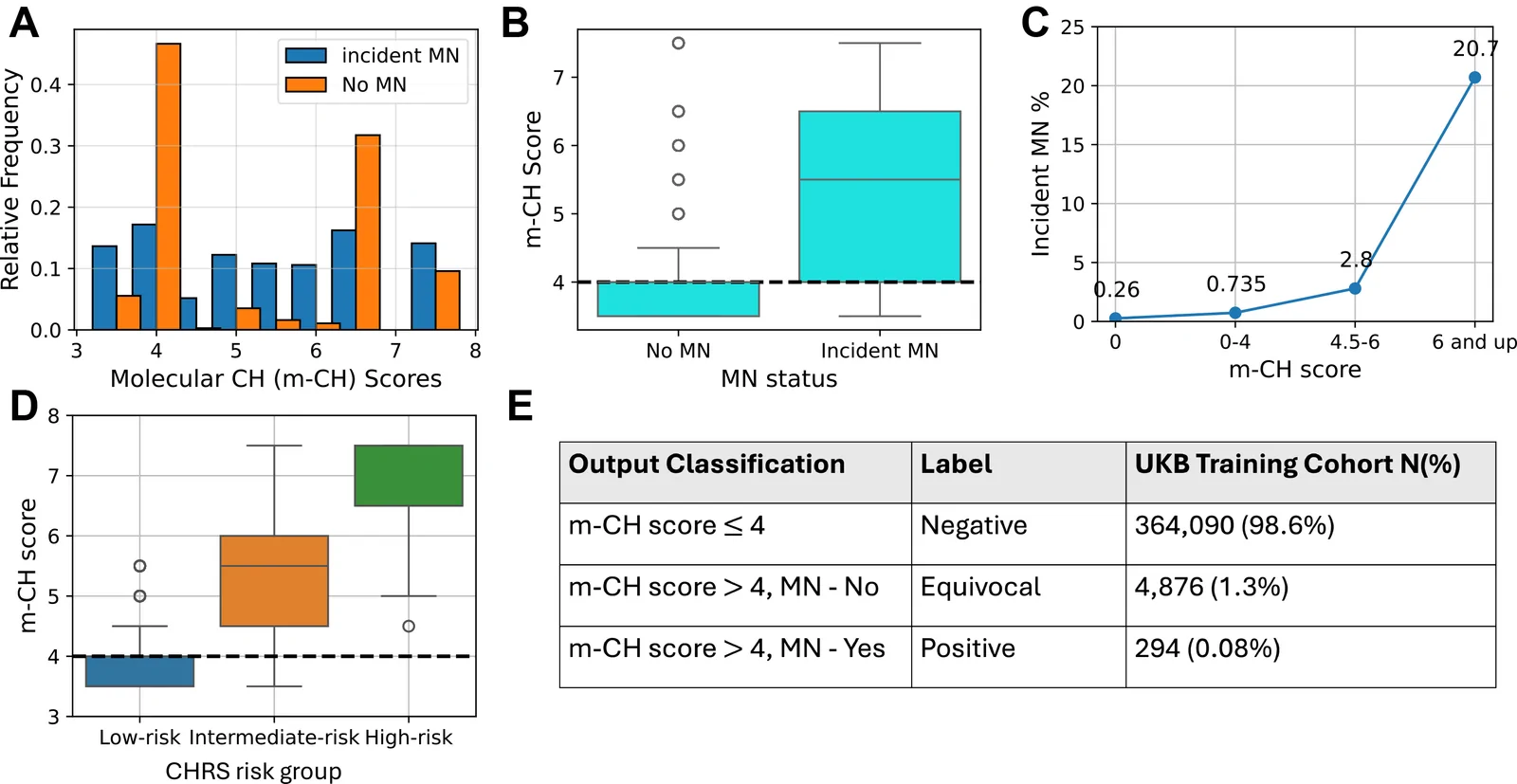
**m-CH score distribution in UKB training set.** (A) Distribution of m-CH scores for CH-positive individuals with and without incident MN. Each bar represents the proportion of individuals with that score. (B) m-CH score distribution for individuals with and without incident MN in UKB. (B) Percentage of individuals with incident MN plotted as a function of binned m-CH scores with values annotated above each data point. (D) Distribution of m-CH scores by CHRS risk groups. For parts B and D, box limits represent IQR and error bars represent the range of the data excluding outliers (beyond 1.5∗IQR). A horizonal line (dashed) is drawn at m-CH score of 4 which was selected as the threshold for MN distribution. (E) Table of ground truth output classifications for the UK Biobank training dataset. CHRS = clonal haematopoiesis risk score. m-CH = molecular clonal haematopoiesis. MN = myeloid neoplasia.

From an initial input feature list (Supplementary Fig. S4A), we eliminated highly correlated CBC parameters (Supplementary Fig. S4B), features non-essential to model performance (Supplementary Fig. S4D), and mean reticulocyte volume, mean sphere cell volume, immature reticulocyte count, and mean corpuscular haemoglobin as features with inconsistent availability across datasets. We verified that dropping these features did not significantly alter model performance (Supplementary Table S3). The pre-sequencing risk model (PRISM) was derived by retraining the tree-based model on UKB training data using the following final set of input features: age; absolute red blood cell, lymphocyte, monocyte, neutrophil, eosinophil, reticulocyte, and platelet counts; haemoglobin; RDW; platelet crit; MCV; and giant platelets. Summary values for each feature are provided in Supplementary Table S4.

We then used the derived PRISM model to make predictions against ground truth labels for 3 available validation cohorts. Confusion matrices are shown in Supplementary Fig. S7, and model performance characteristics are summarised in Supplementary Table S5. As intended, we achieved the highest AUC for the positive class in the UKB test set (0.89, 95% CI 0.85–0.93), and a modest performance was observed for negative (AUC 0.69, 0.68–0.70) and equivocal classes (0.59, 0.57–0.61; Fig. 2A). Sensitivity for ground truth label predictions was also maximised for the positive class (0.73, 95% CI 0.61–0.81; Supplementary Table S5). Of the 73 individuals in the UKB test set with m-CH score of more than 4 who developed incident myeloid neoplasia (Table 1), 53 (72.6%) were characterised by PRISM as positive, 17 (23.3%) as equivocal, and three (4.1%) as negative (Supplementary Fig. S7A). Notably, the three individuals misclassified as negative had normal blood counts and were younger than 60 years, reflecting an unavoidable limitation of single time-point CBC-based screening.

**Fig. 2:**
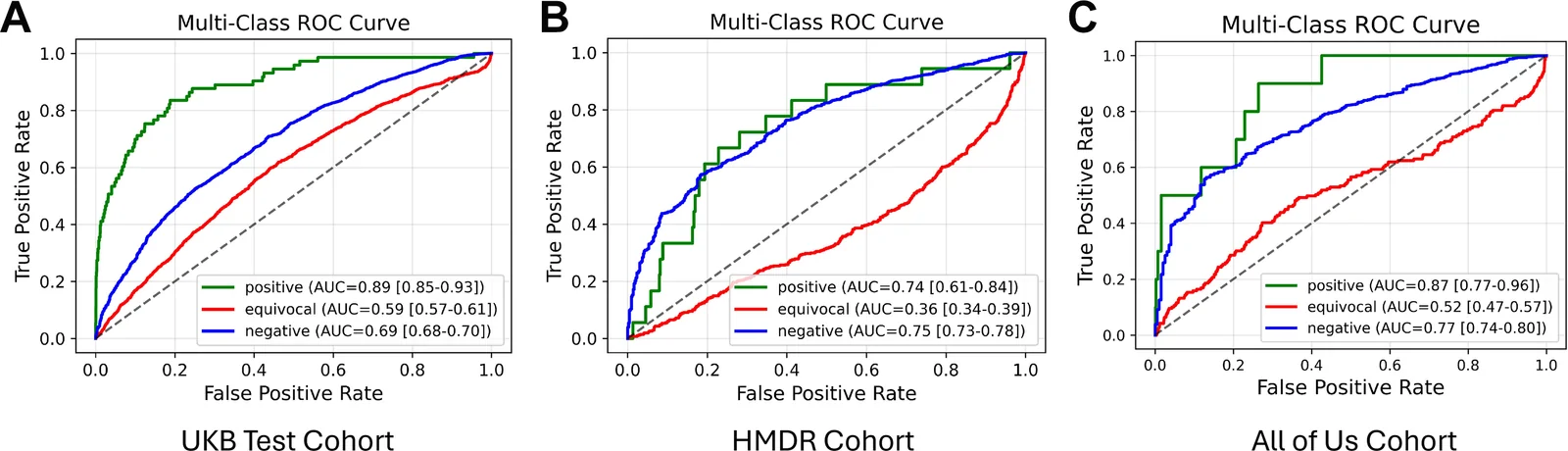
**Pre-NGS screening model (PRISM) performance evaluation.** One-versus-rest ROC curves for positive (green), equivocal (red), and negative (blue) PRISM predicted classes for the UKB test cohort (A), HMDR cohort (B), and All of US cohort (C). AUC for each class is annotated as AUC (95% CI) on the bottom right of each graph with shaded areas representing pointwise 95% CIs. AUC = area under the ROC curve. HMDR = Dana-Farber Cancer Institute Hematologic Malignancy Data Repository. ROC = receiver operating characteristic, UKB = UK Biobank.

The overall negative predictive value (NPV) for PRISM was high (0.993, 95% CI 0.992–0.993; Supplementary Table S5) highlighting the usefulness of PRISM negative class labels to define a population unlikely to benefit from molecular screening for clonal haematopoiesis. As a pre-screening decision aid designed to maximise positive recall, PRISM is intentionally permissive of false positives, resulting in an expectedly low positive predictive value (PPV) for positive class assignments (Supplementary Table S5).

We next tested PRISM generalisability to external clinical and population datasets. The HMDR clinical cohort (n = 2233) had an average age of 63 years and is composed of individuals evaluated in haematology-oncology clinics. Baseline cytopenia rates were higher for HMDR, with CCUS diagnosis in 602 (27%) versus 0.5% of UKB. Despite shorter median follow-up time of 4.9 years (range 0–9.9 years), the overall myeloid neoplasia incidence was higher in HMDR (0.9%) compared to UKB (0.36%), Table 1. Even with this distinction in cohort composition, PRISM correctly assigned positive class labels to 16 (89%) of 18 individuals possessing positive ground truth labels (Supplementary Fig. S7B), corresponding to an AUC of 0.74 (95% CI 0.61–0.84; Fig. 2B) and a sensitivity of 0.89 (95% CI 0.67–0.97) for the positive class (Supplementary Table S5). Both NPV and PPV are affected by the rarity of high-risk clonal haematopoiesis within the population (Supplementary Table S5), Thus, overall NPV remained high at 0.968 (95% CI 0.943–0.981) in HMDR, and, although PPV remained low, the higher prevalence of high-risk clonal haematopoiesis (Table 1) resulted in a higher PPV in HMDR compared with UKB (Supplementary Table S5). Although a proportion of individuals with non-clonal cytopenias and CCUS were classified as negative by PRISM, indicating they would have been excluded from molecular profiling for clonal haematopoiesis, it is notable that the majority of cytopenic individuals receive a positive class label by PRISM (Supplementary Table S6).

All of US, a population cohort of 14,674 individuals, had a median follow-up time of 2.1 years (0.5–4.0). While the age distribution of All of US was similar to UKB, rates of CCUS (n = 183 [1.25%]) and myeloid neoplasia incidence (n = 154 [1.05%]) were higher, which likely reflects medical centre-based study recruitment (Table 1). PRISM had an AUC of 0.87 (95% CI 0.77–0.96; Fig. 2C) and perfect sensitivity for the positive class, with the caveat that only 10 PRISM positive cases were included in the All of US cohort. The overall PRISM NPV was 0.998 (95% CI 0.997–0.999; Supplementary Table S5). The AUC for the negative class improved for both external cohorts. However, across cohorts, equivocal predictions were less reliable reflecting PRISM’s tendency to classify cases as frankly positive or frankly negative.

To confirm the exclusion of high-risk clonal haematopoiesis from populations labelled as PRISM negative, we benchmarked PRISM class predictions against CHRS-determined risk.^9^ In the UKB test set, positive class assignment captures 42 (100%) of 42 CHRS high-risk individuals and 259 (56%) of 462 intermediate-risk individuals (Supplementary Table S6). Meanwhile, negative class assignments were enriched among individuals without clonal haematopoiesis (47,894 [55.4%] of 86,429) and with low-risk clonal haematopoiesis (2158 [40.1%] of 5383; Fig. 3A). False positives, defined here as individuals with positive class labels who did not have high-risk clonal haematopoiesis by CHRS, were minimal. 651 (12%) of 5383 individuals with low-risk clonal haematopoiesis and 9415 (10.9%) of 86,429 without clonal haematopoiesis were erroneously classified as positive by PRISM (Fig. 3A).

**Fig. 3:**
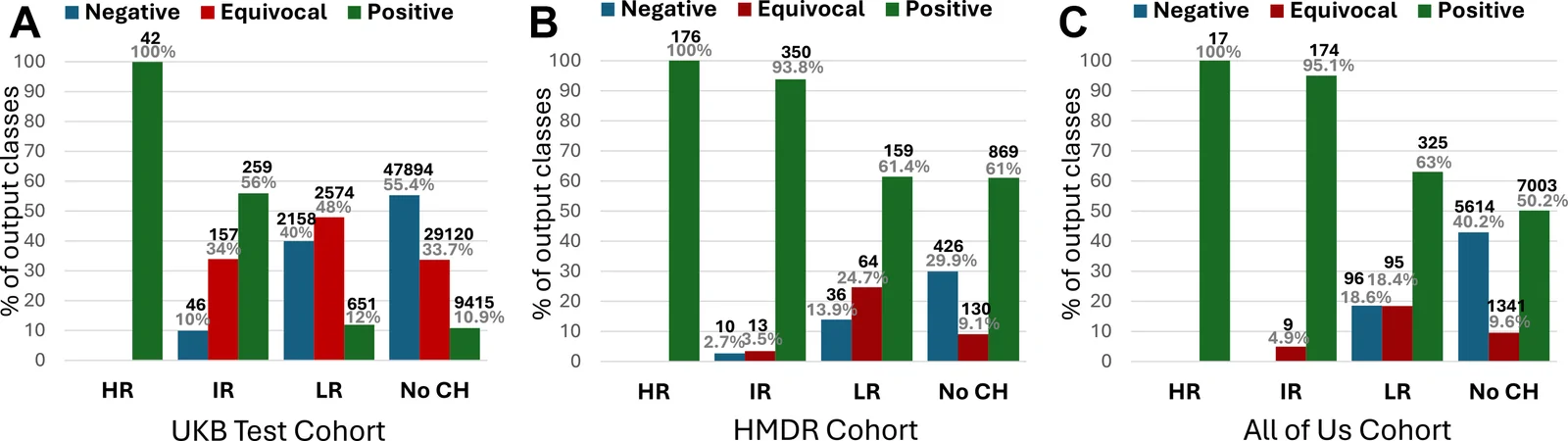
**PRISM classifications compared to post-next generation sequencing risk predictions by CHRS.** Distribution of PRISM class assignment positive (green), negative (blue) and equivocal (red) according to post-next generation sequencing CHRS risk groups for individuals in the UK Biobank test cohort (A), HMDR cohort (B), and All of US cohort (C). CHRS risk groups are labelled to indicate high-risk (HR), intermediate-risk (IR), low-risk (LR) and individuals without for positive versus negative class labels (no CH). Bars are annotated with the N (%) for each PRISM class. CHRS = clonal haematopoiesis risk score. HMDR = Dana-Farber Cancer Institute Hematologic Malignancy Data Repository.

Positive class labeling captured 176 (100%) of CHRS-defined high-risk and 350 (of 373, 93.8%) of CHRS-defined intermediate risk individuals in the HMDR, showing benchmarking was equally good in a clinical setting (Fig. 3B, Supplementary Table S6). Likewise, all 17 (100%) high-risk and 174 (of 183, 95.1%) intermediate-risk clonal haematopoiesis carriers in All of US were classified as positive by PRISM (Fig. 3C). We note that false positive classification was higher in both external cohorts, owing to higher rates of baseline cytopenias relative to the UKB (Table 1).

We separately evaluated PRISM positive class label capture of high-risk clonal haematopoiesis as defined for cytopenic subpopulations of each cohort using the clonal cytopenia risk score (CCRS)^10^ in the cytopenic fraction of the UKB test cohort. Despite non-overlapping risk definitions and class labels for the CHRS and CCRS (Supplementary Fig. S8A), CHRS and CCRS risk classifications similarly demonstrate enrichment for clonal haematopoiesis with high-risk molecular features (defined by m-CH score >4) in the high-risk strata (Supplementary Fig. S8B and C). Moreover, 18 of (95%) 19 of CCRS-defined high-risk CCUS were labelled as PRISM positive (Supplementary Fig. S8D). The one (5%) remaining CCRS-defined high-risk CCUS case was labelled as equivocal (Supplementary Fig. S8D) and reassuringly did not progress to myeloid neoplasia.

We compared PRISM performance to simple clinical indicators, such as age, cytopenia, high MCV, and high RDW. PRISM positive classification was superior, displaying a sensitivity of 1.0 for high-risk clonal haematopoiesis identification in UKB (Supplementary Fig. S9), corresponding to one high-risk clonal haematopoiesis case identified for every 247 individuals identified as positive by PRISM (number needed to screen).

To have efficacy as a pre-screening tool, individuals with clonal haematopoiesis who develop incident myeloid neoplasia should be classified as positive by PRISM. We tested this by examining the proportion of individuals with clonal haematopoiesis who developed incident myeloid neoplasia for each PRISM class (Fig. 4A). Of 114 such individuals, PRISM classified 72 (63%) as positive, 29 (25%) as equivocal, and 13 (11%) as negative (Fig. 4A). Among cases misclassified as negative, post-sequencing CHRS classification would have labelled them as low risk (12 of 13) or intermediate risk (1 of 13). Positive class predictions improved for HMDR and All of US cohorts, where 16 (89%) of 18 individuals were correctly classified as positive in HMDR and 18 (100%) of 18 in All of US (Fig. 4A).

**Fig. 4:**
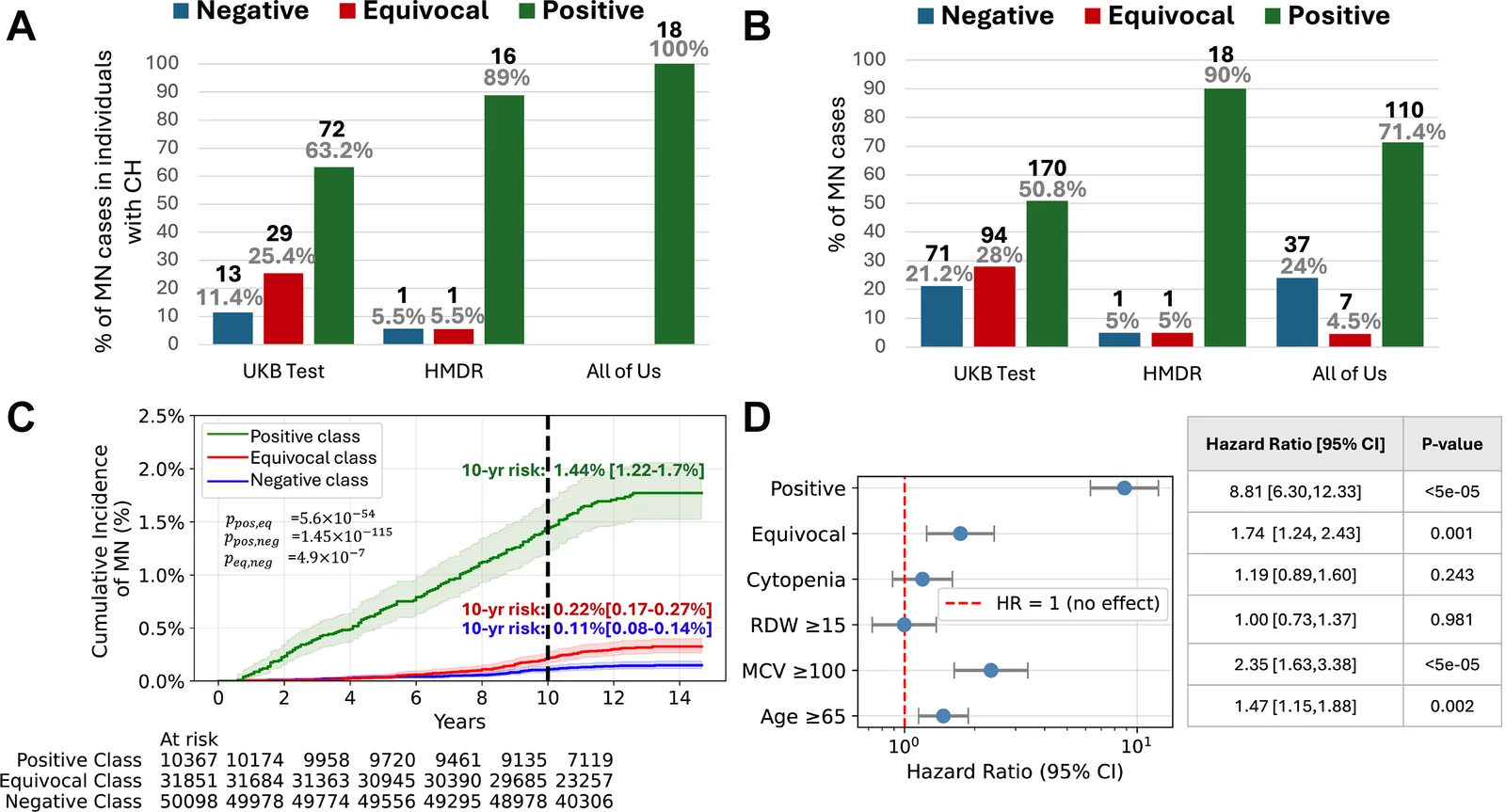
**Incidence of MN by PRISM classification.** (A) Distribution of PRISM classes for individuals with clonal haematopoiesis who develop incident MN. (B) Distribution of PRISM classes for all individuals with incident MN regardless of CH status by validation cohort. For both parts A and B, bars represent positive (green), negative (blue), and equivocal (red) PRISM classes. (C) Cumulative incidence of MN for individuals in UK Biobank test cohort labelled positive (green), negative (blue) and equivocal (red) by PRISM. Observation time (years) reflects censorship at death or loss to follow-up. Log-rank test was used for cumulative incidence between curves. (D) Hazard ratios (95% CI) for incident MN were estimated using multivariate Cox proportional hazard model for each PRISM output class, age greater than or equal to 65 (age ≥ 65 years), and select blood count features: MCV, RDW, and presence of cytopenia. CH = clonal haematopoiesis. MCV = mean cellular volume. MN = myeloid neoplasia. RDW = red cell distribution width.

In each cohort, a minority of incident myeloid neoplasia events occur among individuals without clonal haematopoiesis detected at baseline (Supplementary Table S7), likely representing myeloid neoplasias evolving from clonal haematopoiesis with VAF of less than 2% at baseline or myeloid neoplasia events driven by other molecular drivers (e.g., de-novo acute myeloid leukaemia with *NPM1* mutations or chromosomal abnormalities). Still, most incident myeloid neoplasia cases are classified as positive or equivocal, specifically 264 (79%) of 335 individuals with incident myeloid neoplasia in UKB, 19 (95%) of 20 In HMDR, and 117 (76%) of 154 in All of US (Fig. 4B). Cases with incident myeloid neoplasia labelled negative by PRISM include individuals without clonal haematopoiesis at baseline or individuals who had low-risk clonal haematopoiesis. For instance, in the UKB, of the 71 PRISM negative individuals who developed incident myeloid neoplasia, 58 (8w%) had no clonal haematopoiesis at baseline, 12 (17%) were low risk by CHRS, and 1 (1%) was intermediate risk by CHRS (Supplementary Table S8).

Myeloid neoplasia risk is distinct for each PRISM class. In the UKB, the Kaplan–Meier estimated 10-year cumulative incidence of myeloid neoplasia was 1.44% (95% CI 1.22–1.70) for positive, 0.11% (0.08–0.14) for negative, and 0.22% (0.17–0.27) for equivocal classes (Fig. 4C). For positive versus negative class labels, the hazard ratio for incident myeloid neoplasia was 8.81 (95% CI 6.3–12.33), making PRISM positive predictions a more robust predictor of myeloid neoplasia outcomes in multivariate cox proportional hazard models than age, cytopenia, MCV, or RDW (Fig. 4D).

Clonal haematopoiesis is independently associated with cardiovascular disease outcomes which significantly influence clonal haematopoiesis-associated mortality.^1^^,^^21^ As PRISM is intended to determine need for subsequent sequencing for clonal haematopoiesis, we evaluated PRISM class associations with cardiovascular disease to ensure cardiovascular disease events were not enriched in the PRISM negative subpopulation. The cumulative incidence of cardiovascular disease in UKB participants varied predictably by PRISM classification, with the Kaplan–Meier estimated 10-year risks equal to 15.7% (95% CI 15–16.4) for positive, 13.4% (13–13.8) for equivocal, and 4.6% (4.4–4.8) for negative classes (Fig. 5A). Absolute cardiovascular disease event rates were concentrated among PRISM positive and equivocal classes for UKB participants (Fig. 5B). Cardiovascular disease risk for PRISM positive class predictions was comparable to age 65 years or older in multivariable hazard models (Fig. 5C).

**Fig. 5:**
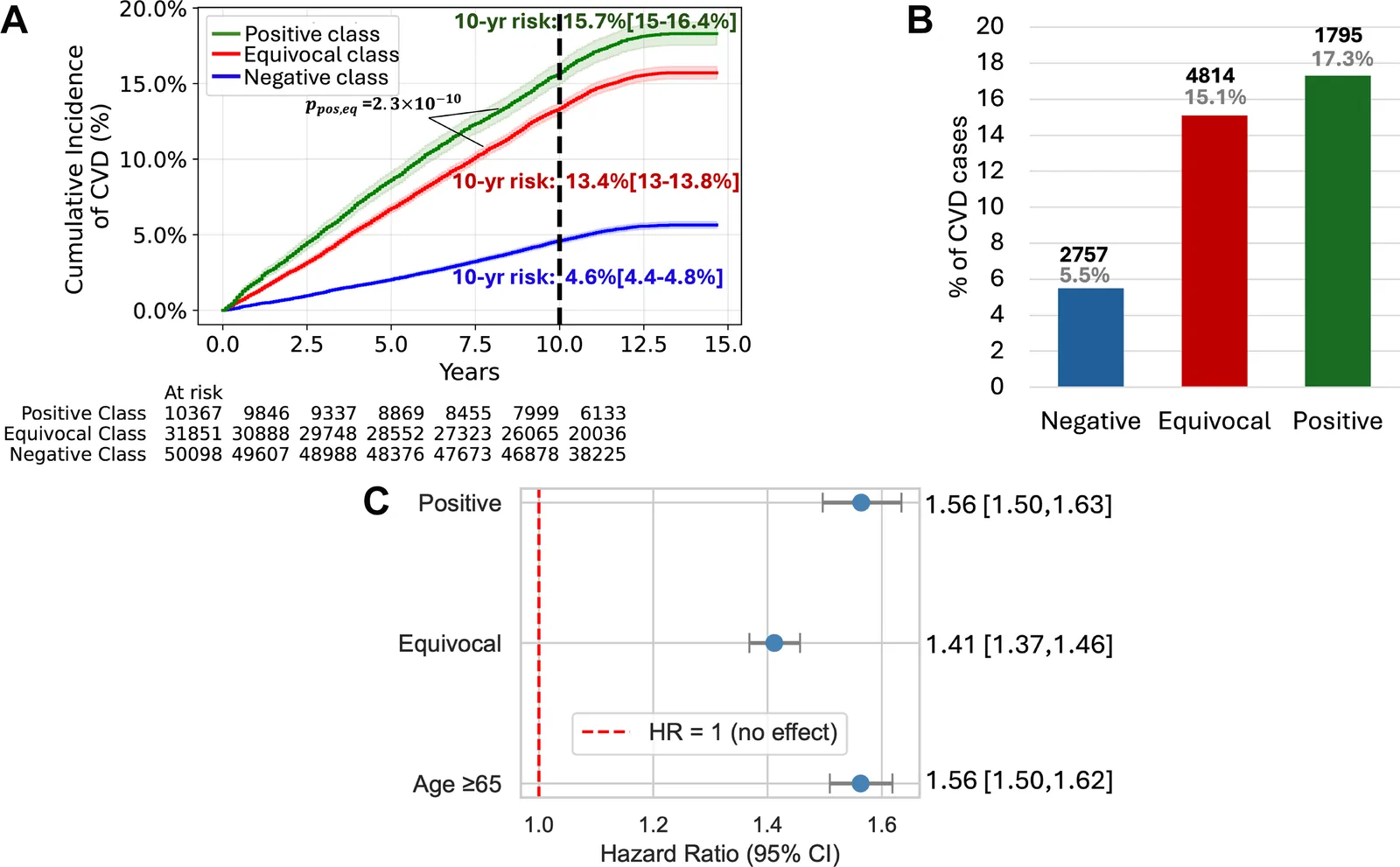
**Cumulative incidence of CVD outcomes by PRISM classification.** CVD cases are defined as incident diagnoses of myocardial infraction, angina, atherosclerosis, ischaemic heart failure, ischaemic stroke and other ischaemic CVDs. (A) Cumulative incidence of CVD among individuals classified as positive (green), negative (red), and equivocal (blue) by PRISM in the UK Biobank test cohort. Comparison between class labels was performed using log-rank test with censoring at death or loss to follow-up. (B) Bar graph showing percentage of incident CVD cases by PRISM class. Bars are annotated with the number of cases (%). (C) Hazard ratios (95% CI) for incident CVD risk estimated using multivariate Cox proportional hazard model for PRISM output classes and age ≥ 65 years. CVD = cardiovascular disease.

We summarise the intended use for PRISM as a pre-screening tool in Fig. 6 and provide example cases in the Supplemental materials. Positive classification by PRISM flags individuals for follow-up sequencing, justified by a relatively high likelihood of having clinically relevant clonal haematopoiesis. An example case indicates an individual with a positive result from PRISM evaluation of CBC data with clinical data indicating a finding of high-risk CCUS and progression to myeloid neoplasia within 5 years of CCUS diagnosis. By contrast, high-risk clonal haematopoiesis is characteristically absent from populations labelled negative by PRISM in all three cohorts evaluated. Therefore, individuals who pre-screen negative with PRISM by virtue of being unlikely to have clinically relevant clonal haematopoiesis lack an indication for molecular screening for clonal haematopoiesis absent other compelling clinical indicators. The equivocal class represents a group for which decisions about sequencing for clonal haematopoiesis cannot be made using single time-point CBC information in isolation. Although further validation is necessary before clinical use, a PRISM web calculator is provided in the supplement to facilitate usage in clinical research.

**Fig. 6:**
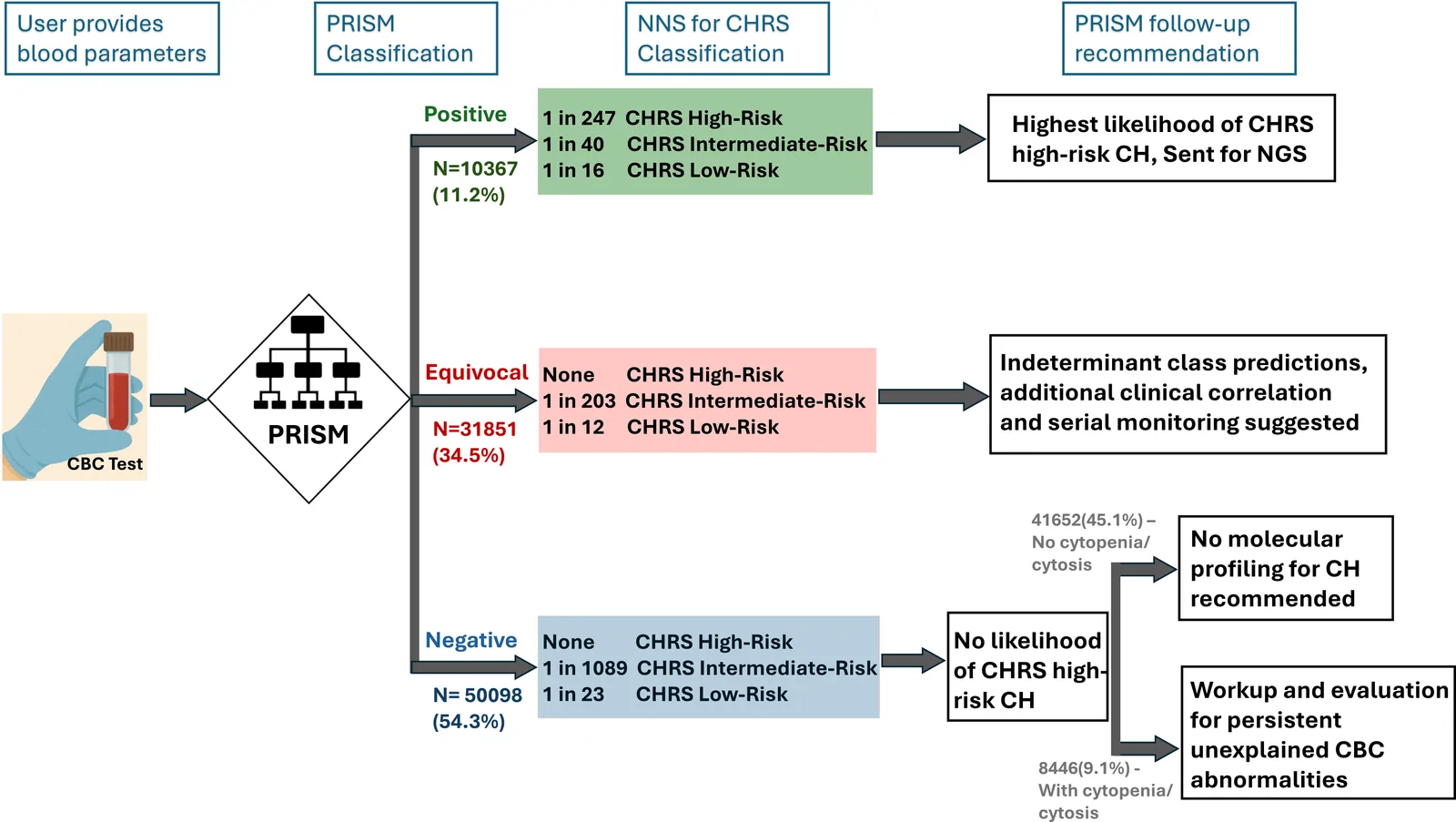
**Proposed usage of PRISM in clinical research settings.** PRISM predictions are intended to inform downstream decision-making molecular profiling for clonal haematopoiesis with sequencing assays. Users input complete blood count parameters into the PRISM model and are provided with outputs of positive, equivocal, or negative for that individual. Those screening as positive have the highest risk of having high-risk clonal haematopoiesis as defined by the CHRS and would be an ideal population for subsequent molecular profiling. Individuals screening negative are unlikely to have high-risk clonal haematopoiesis and should not receive follow-up molecular profiling absent another compelling indication. The equivocal class represents individuals for whom single time-point testing is insufficient to evaluate the potential benefit of follow-up sequencing; serial assessments or further clinical correlation will be necessary. All numbers shown in this schematic are derived from PRISM prediction and benchmarking in UKB test cohort. CBC = complete blood cell count. CHRS = clonal haematopoiesis risk score. NGS = next generation sequencing. NNS = number needed to screen.

## Discussion

Identification of populations enriched for high-risk clonal haematopoiesis and clinically significant risk of incident myeloid neoplasia is essential for the efficient and cost-effective development of clonal haematopoiesis monitoring and myeloid neoplasia prevention strategies. Here, we report CBC parameters associated with progression of high-risk clonal haematopoiesis to myeloid neoplasia and incorporate them into PRISM, a novel pre-sequencing model that establishes risk thresholds for the use of molecular profiling for clonal haematopoiesis. PRISM classifies adults as belonging to 3 classes: positive—likely to benefit from sequencing for clonal haematopoiesis due to relatively high-risk for myeloid neoplasia; negative—unlikely to benefit from sequencing for clonal haematopoiesis due to low likelihood of clinically relevant clonal haematopoiesis; and equivocal—individuals for whom single timepoint CBC predictions are not possible. True at-risk individuals are routinely identified by positive class labels, with 100% recall of CHRS high-risk cases in the positive class and an AUC of 0.83 across validation cohorts.

Cohort characteristics significantly influenced PRISM’s false positive rate, with a higher proportion of cases of low-risk clonal haematopoiesis labelled as positive by PRISM in populations with baseline CBC abnormalities (Supplementary Fig. S7). This reflects an intentional emphasis on maximising true positives and minimising false negatives. PRISM classification does not define the presence or absence of clonal haematopoiesis, but rather flags with a positive label, potentially high-risk individuals for further screening with molecular assays. In clinical use, false positives are expected to be resolved with follow-up sequencing. Variation in observation times across cohorts is also a limitation of our analysis. As longer validation allows more time for incident myeloid neoplasia, the short observation times for HMDR and All of US may result in inaccurate ground truth labels and inaccurate assessment of model performance. To mitigate variations in observation times across cohorts, we benchmarked PRISM against single time point metrics of clonal haematopoiesis risk including the CHRS, CCRS, and standard clinical predictors of high-risk clonal haematopoiesis.

Despite cohort-level differences including indications for CBC acquisition and sequencing methodology, our findings validate PRISM as a discriminator of populations enriched for high-risk clonal haematopoiesis from populations without high-risk clonal haematopoiesis. In all validation settings, CHRS high-risk clonal haematopoiesis is completely captured by positive class labeling and intermediate-risk clonal haematopoiesis rarely receives a negative class label. Thus, we confidently interpret negative class predictions by PRISM as “ruling out” potential high-risk clonal haematopoiesis cases. Used as intended (Fig. 6), deferring sequencing for individuals with negative class predictions would result in markedly reduced detection of low-risk clonal haematopoiesis (14–40% fewer cases sequenced) and spare many without clonal haematopoiesis from receiving an unnecessary molecular evaluation (30–55% fewer no clonal haematopoiesis cases sequenced, Fig. 3). In a general population like the UKB, PRISM use results in detection of 1 high-risk clonal haematopoiesis case for every 247 individuals recommended for sequencing for clonal haematopoiesis (Fig. 6) compared to 1 high-risk clonal haematopoiesis case for every 2500 people screened in a strategy targeting all adults.

PRISM defines a heterogenous equivocal class that may be comprised of (1) individuals with intermediate-risk clonal haematopoiesis with mostly normal baseline blood counts; (2) individuals with abnormal blood counts who do not have clonal haematopoiesis; and (3) individuals with abnormal blood counts who have low-risk clonal haematopoiesis. Reflected by the low AUC for this class label, our model is unable to discriminate risk based on CBC data alone for individuals screening as equivocal with PRISM. Ongoing work to incorporate additional prognostic features including peripheral blood cell morphology, changes in blood counts over time, chronic comorbidity, or cytotoxin exposures may improve equivocal class labeling. We emphasise that PRISM, as presented, is not intended as tool to diagnose high-risk clonal haematopoiesis, but rather is meant to augment and complement clinical judgment on the use of molecular profiling for clonal haematopoiesis Importantly, individuals who screen as equivocal but have persistent and unexplained blood count abnormalities continue to have an inherent primary indication for further haematological malignancy investigation, often inclusive of molecular profiling.^24^

There are several potential clinical and clinical research applications of PRISM. As clinical assays for clonal haematopoiesis become more widely available, haematology clinics specialising in the diagnosis and management of clonal haematopoiesis,^25^ may find that PRISM negative classifications reassuring to patients who will not have high-risk clonal haematopoiesis at the time of assessment. Alternatively, in allogeneic stem cell transplant where donor-engrafted clonal haematopoiesis is a rare complication occurring exclusively among donors with high-risk clonal haematopoiesis profiles,^26^^,^^27^ PRISM evaluation donor CBCs may identify donors at-risk of harboring high-risk clonal haematopoiesis who should be screened with sequencing and excluded from the donor pool.

Clonal haematopoiesis is associated with non-malignant health outcomes, including, cardiovascular diseases.^2^^,^^28^ Given a proposed shared biology directing clonal haematopoiesis-associated malignant and non-malignant outcomes,^29^ we evaluated whether PRISM negative class labeling would inadvertently exclude individuals with clonal haematopoiesis-associated elevations in Crisk^30^ from molecular profiling for clonal haematopoiesis. Reassuringly, cardiovascular disease risk was lowest among PRISM negative individuals, confirming that deferment of sequencing for clonal haematopoiesis in this population would not disproportionately exclude evaluation of clonal haematopoiesis-associated incident cardiovascular disease. Direct modeling of cardiovascular disease outcomes is warranted, particularly if viable strategies to target clonal haematopoiesis to prevent cardiovascular disease emerge.^24^^,^^30^

PRISM derivation is conceptually aligned with previous works that derived machine learning and regression-based models to predict diagnosis of myeloid neoplasia,^9^, ^10^, ^11^^,^^14^^,^^31^^,^^32^ and clonal haematopoiesis.^33^ Rather than predict specific clonal haematopoiesis genotypes,^33^ we focused on pre-sequencing prediction of high-risk clonal haematopoiesis likely to result in myeloid neoplasia, towards development of population-level screening strategies with public health relevance. The consistent identification of morphology-related CBC parameters as strong predictors of myeloid neoplasia risk is of interest. Our ability to identify and validate morphology-related CBC parameters was however limited by inconsistent annotation of these features between datasets. Routine recording of extended CBC parameters in clinical and research datasets, and direct analysis of peripheral blood smear images are important future efforts that will resolve associations between haematopoietic cell morphologies, clonal haematopoiesis molecular features, and clinical outcomes.

Deriving CBC-based predictors using single timepoint data has inherent limitations. CBC value fluctuations due to illness, hydration status, and nutritional state are often revealed by serial CBC assessment. Classification of a minority of individuals as PRISM negative despite the presence of clonal haematopoiesis and ultimate progression to myeloid neoplasia likely reflects this limitation. As blood count abnormalities to precede myeloid neoplasia transformation,^34^ serial sampling of blood counts and PRISM assessment is likely to capture additional at-risk individuals.

Though we’ve used 3 distinct cohorts for validation, PRISM may not be generalisable to all populations and clinical contexts. Specialised contexts such as chemotherapy exposure, inherited bone marrow failure conditions, or haemoglobinopathies will have markedly distinct haematopoietic evolutionary dynamics, genotype distribution of clonal haematopoiesis, and thus rates of clonal haematopoiesis transformation.^25^^,^^35^ Therefore, context-specific adjustments to the levels of feature importance and consideration of alternative predictive features will be necessary in clinical scenarios characterised by particularly strong intrinsic or extrinsic clonal haematopoiesis selection pressures. Derivation of PRISM in the UKB also limits our ability to generalise to ancestrally diverse populations given the predominantly European ancestry of the cohort and recent observations of ancestry-specific effects on clonal haematopoiesis prevalence.^36^ Lastly, while useful for capturing non-linear and complex covariate-outcome relationships, machine learning models may obscure specific covariate contributions to model performance and do not account for the relevance of covariate relationships in distinct clinical contexts. Context-specific optimisation and validation of PRISM in diverse cohorts is an important next step for model development.

Early-stage cancer diagnosis has proven benefit in reducing disease-related mortality. For myeloid neoplasias, identifying subpopulations that may benefit from screening for clonal haematopoiesis remains a major challenge. As a pre-sequencing screening tool, PRISM addresses this unmet need and provides a tool that can direct molecular profiling towards subgroups enriched for high-risk clonal haematopoiesis while limiting unnecessary sequencing in subpopulations unlikely to have clinically relevant clonal haematopoiesis. In this way, PRISM is an initial step towards a more judicious use of molecular diagnostics in undifferentiated populations, sparing a significant fraction of lower risk individuals from the psychosocial and financial hazards of overdiagnosis of clonal haematopoiesis.^16^

## Contributors

LDW and RN conceived the study. RN performed initial model derivation, all statistical analysis, generated table and figures and wrote the manuscript. LW oversaw the analysis and performed critical edits to analysis plan, statistical approach, tables, figures and manuscript text. SS, ML, RCL and DJD provided HMDR cohort. NNK and HU provided statistical review and technical validation of model derivation and performance. AB provided access to All of US cohort for external validation and KZ and YP performed validation. AB, PN, CV, MU, performed clonal haematopoiesis variant calls in UKB. All authors provided critical input to data interpretation, preparation of manuscript, and read and approved the final version of the manuscript for publication. LDW and RN had access to and validated all study data.

## Data sharing statement

Data from UK Biobank and All of US are available to researchers via application. De-identified data from HMDR are accessible to interested researchers via request to corresponding author. Relevant codes and scripts used for data analysis and model derivation are available at https://github.com/rnandi11/PRISM_CH.

## Declaration of interests

LDW reports advisory board/consulting for Vertex, Sobi, unrelated to the present work. RCL reports consulting for Qiagen, and Novartis, and research funding from bluebird bio (now Genetix), unrelated to the present work. SS report institutional research funding from Syndax. PN reports research grants from Allelica, Amgen, Apple, Boston Scientific, Cleerly, Genentech/Roche, Ionis, Novartis, and Silence Therapeutics, institutional research funding from Novo Nordisk, personal fees from ARINA, Allelica, Amgen, Apple, AstraZeneca, Bain Capital, Blackstone Life Sciences, Bristol Myers Squibb, Broadview Ventures, Creative Education Concepts, CRISPR Therapeutics, Eli Lilly & Co, Esperion Therapeutics, Foresite Capital, Foresite Labs, Genentech/Roche, GV, HeartFlow, Incyte, Magnet Biomedicine, Merck, Novartis, Novo Nordisk, TenSixteen Bio, Tourmaline Bio, and Ursa Medicines, equity in Amgen, Bolt, Candela, Mercury, MyOme, Parameter Health, Preciseli, and TenSixteen Bio, royalties from Recora for intensive cardiac rehabilitation, employment at Amgen, and spousal employment at Vertex Pharmaceuticals, all unrelated to the present work. ML reports advisory board/consulting for AbbVie, Amgen, Astra Zeneca, Autolus, Enliven, Jazz, KITE, Menari Stemline, Merck, Novartis, Pfizer, Servier, Syndax unrelated to the present work. DJD reports research grants from Abbvie, Blueprint, Cogent, and Novartis Pharmaceuticals, participation on a Data Safety Monitoring Board or Advisory Board for Daiichi-Sankyo. Mt Sinai MPN Consortium and ad hoc consultation fees from Amgen, Autolos, Agios, Blueprint, Forty-Seven, Gilead, Incyte, Jazz, Novartis, Pfizer, Servier, and Takeda. RN, KZ, YP, NNK, CV, MMU, HU and AB declare no competing interests.
