# Supplementary material for: Derivation of a blood count-based machine learning model to prioritise sequencing for high-risk clonal haematopoiesis: a retrospective cohort study
Source: eClinicalMedicine. 2026 Sep 22;100:104238. doi: 10.1016/j.eclinm.2026.104238 (PMC13659614; doi:10.1016/j.eclinm.2026.104238)
Supplement: Supplementary file 1 [file mmc1.docx]

**Supplementary Materials**

**List of Content in Supplementary Materials**

**Derivation of a blood count-based machine learning model to prioritize sequencing for high-risk clonal hematopoiesis: a retrospective cohort study.**

Nandi R., et al.

|  | Page # |
| --- | --- |
| Supplemental Tables | |
| List of Supplementary Tables | 1 |
| Table S1: ICD10 codes used in study | 2 |
| Table S2: CHRS features utilized to determine Molecular CH (m-CH) Score | 3 |
| Table S3: Model performance before and after feature elimination | 4 |
| Table S4: Summary of features utilized for PRISM derivation | 5 |
| Table S5: PRISM performance characteristics | 6 |
| Table S6: Characteristics of individuals in each PRISM output class in the UKB test cohort and HMDR | 9 |
| Table S7: Myeloid neoplasia (MN) outcomes in each cohort by CHRS risk group assignment | 10 |
| Table S8: Characteristics of individuals with incident MN in each PRISM output classes in UKB test cohort | 11 |
|  |  |
| Supplemental Figures | |
| List of Supplementary Figures | 12 |
| Figure S1: Consort diagram for the UKB training and test cohorts | 13 |
| Figure S2: Consort diagram for the HMDR cohort | 14 |
| FigureS3: Consort diagram for the All of Us cohort | 15 |
| Figure S4: Feature and Architecture Selection. | 16 |
| Figure S5: Platelet Distribution Width | 19 |
| Figure S6: Mutation Profiles of Cohorts | 20 |
| Figure S7: Confusion Matrices of Cohorts | 21 |
| Figure S8: PRISM benchmarking against CCRS | 22 |
| Figure S9: PRISM benchmarking against blood count and age | 23 |
|  |  |
| Example PRISM use cases randomly selected from the HMDR clinical cohort | 24 |

**Supplementary Tables**

|  | Page # |
| --- | --- |
| List of Supplementary Tables |  |
| Table S1: ICD10 codes used in study | 2 |
| Table S2: CHRS features utilized to determine Molecular CH (m-CH) Score | 3 |
| Table S3: Model performance before and after feature elimination | 4 |
| Table S4: Summary of features utilized for PRISM derivation | 5 |
| Table S5: PRISM performance characteristics | 6 |
| Table S6: Characteristics of individuals in each PRISM output class in the UKB test cohort and HMDR | 10 |
| Table S7: Myeloid neoplasia (MN) outcomes in each cohort by CHRS risk group assignment | 11 |
| Table S8: Characteristics of individuals with incident MN in each PRISM output classes in UKB test cohort | 12 |

| **Table S1: ICD-10 codes used in study** | |
| --- | --- |
| **Myeloid Neoplasms (MN) outcome codes** | |
| Myelodysplastic Syndrome (MDS) | D460, D461, D462, D463, D464, D465, D466, D467, D469 |
| Acute Myeloid Leukemia (AML) | C920, C923, C924, C925, C926, C930, C937, C939, C940, C942, C943, C944, C950 |
| Myeloproliferative Neoplasm (MPN) | D45, D473, C946, D474 |
| Chronic and other myelogenous Leukemia | C922, C931, C932, C941, C951, C952, D475, C957, C947, C937, C927, C959, C929 |
| **Cardiovascular Disease (CVD) outcome codes** | |
| Myocardial Infraction | I211, I210, I212, I213, I214, I219 |
| Angina | I209 |
| Other Ischemic CVD | I249, I258, I259 |
| Atherosclerosis | I250, I251, I252, I700, I709 |
| Ischemic Heart Failure | I255 |
| Ischemic Stroke | I630, I631, I632, I633, I634, I635, I639, I672, I638, I693 |

| **Table S2: CHRS features utilized to determine Molecular CH (m-CH) score** | | | | |
| --- | --- | --- | --- | --- |
|  | **CHRS Feature Score** | | | |
| **Prognostic Variable** |  | | | |
|  | **0.5** | **1** | **2** | **2.5** |
| Single *DNMT3A* | Present | Absent |  |  |
| High-risk mutation |  | Absent |  | Present |
| Number of Mutations |  | 1 | ≥2 |  |
| Variant Allele Fraction |  | <0.2 | ≥0.2 |  |
| Weighted feature scores for molecular features from the CHRS were assigned for each individual and the sum was used to determine a molecular CH score (MCH).  Abbreviations: CHRS = clonal hematopoiesis risk score | | | | |

| **Table S3: Model performance before and after feature elimination** | | | | |
| --- | --- | --- | --- | --- |
| **Metric** | **Class** | **Baseline Model** | **Post-Boruta Model** | **Final-Feature Model** |
| **AUC** | Negative | 0.69 | 0.69 | 0.69 |
|  | Equivocal | 0.59 | 0.59 | 0.59 |
|  | Positive | 0.89 | 0.88 | 0.89 |
| **Average AUC** |  | 0.72 | 0.72 | 0.72 |
| **Sensitivity** | Negative | 0.56 | 0.55 | 0.55 |
|  | Equivocal | 0.53 | 0.55 | 0.54 |
|  | Positive | 0.63 | 0.59 | 0.63 |
| **Weighted Sensitivity** |  | 0.56 | 0.55 | 0.55 |
| **Accuracy** |  | 0.56 | 0.55 | 0.55 |
| **Weighted Precision** |  | 0.98 | 0.98 | 0.98 |
| **Weighted F1 Score** |  | 0.72 | 0.70 | 0.70 |
| Performance evaluation table for the baseline model with 22 features, post-Boruta model after dropping 5 features deemed insignificant by Boruta, and the final-feature model fitted on the final set of features after empirical feature elimination. AUCs are the measure of area under the receiver-operator characteristic curves. Sensitivity or recall is the model’s recall rate for predicting ground truth labels. Precision is the ratio of true positive over all predicted positives. F1 score is the harmonic mean of recall and precision. Weighted metrics are the mean value of each class’s metric value weighted by that class’s number of true sample size. | | | | |

| **Table S4. Summary of features utilized for PRISM derivation** | | | | |
| --- | --- | --- | --- | --- |
|  | **UKB Training Cohort** | **UKB Test Cohort** | **HMDR** | **All Of Us** |
| **Age in years** | 58.3 (50.6, 63.7) | 58.3 (50.6, 63.7) | 65.8 (53.0, 75.3) | 57.2 (42.2, 67.8) |
| **12 CBC parameters** | | | | |
| **Red blood cell count** | 4.5 (4.2, 4.8) | 4.5 (4.2, 4.8) | 4.1 (3.4, 4.7) | 4.4 (4.0, 4.8) |
| **Hemoglobin** | 14.15 (13.3, 15.0) | 14.1 (13.3, 15.0) | 12.4 (10.4,14.0) | 13.1 (11.9, 14.1) |
| **Mean corpuscular volume** | 91.2 (88.5, 93.9) | 91.2 (88.5, 93.8) | 92.0 (87.6, 97.5) | 90.0 (86.2, 93.4) |
| **Red cell distribution width** | 13.3 (12.9, 13.9) | 13.3 (12.9, 13.9) | 14.2 (13.2, 16.0) | 13.7 (13.0, 14.8) |
| **Platelet count** | 248.3 (213.8, 287.4) | 248.0 (213.0, 287.0) | 199.0 (130.0, 271.0) | 241.0 (199.7, 289.2) |
| **Platelet crit** | 0.23 (0.2, 0.26) | 0.23 (0.2, 0.26) | 0.18 (0.12, 0.24) | 0.45 (0.44, 0.45) |
| **Lymphocyte count** | 1.9 (1.5, 2.3) | 1.9 (1.5, 2.3) | 1.4 (1.0, 2.0) | 1.9 (1.4, 2.8) |
| **Monocyte count** | 0.45 (0.36, 0.57) | 0.45 (0.37, 0.57) | 0.5 (0.4, 0.7) | 0.6 (0.5, 1.0) |
| **Neutrophil count** | 4.0 (3.3, 5.0) | 4.0 (3.3, 5.0) | 3.8 (2.6, 5.4) | 4.4 (2.9, 7.6) |
| **Eosinophil count** | 0.14 (0.1, 0.21) | 0.14 (0.1, 0.21) | 0.14 (0.08, 0.3) | 0.20 (0.10, 0.30) |
| **Reticulocyte count** | 0.06 (0.04, 0.07) | 0.06 (0.04, 0.07) | 0.06 (0.05,0.08) | 0.06 (0.04, 0.09) |
| **Giant Platelets** |  |  |  |  |
| Absent | 284364 (77%) | 71214 (77%) | 2200 (98.5%) | 11825 (80.6%) |
| Present | 84896 (23%) | 21103 (23%) | 33 (1.5%) | 2849 (19.4%) |
| Categorical values are summarized as number and percentage, N (%). Continuous values are summarized as median (interquartile range). Variables shown are the final set of features utilized in deriving the pre-NGS prediction model (PRISM). Values are summarized for the training and test cohorts from the UK Biobank (UKB), the hematologic malignancies data repository (HMDR) clinical cohort, and the All of Us (AOU) population cohort. | | | | |

| **Table S5: PRISM performance characteristics** | | | | | |
| --- | --- | --- | --- | --- | --- |
| **Metric** | **Class** | **UKB Training Cohort**  Estimate [95% CI] | **UKB Test Cohort**  Estimate [95% CI] | **HMDR**  Estimate [95% CI] | **All of Us**  Estimate [95% CI] |
| **AUC** | Positive | 0.94 [0.93-0.95] | 0.89 [0.85-0.93] | 0.74 [0.61-0.84] | 0.87 [0.77-0.96] |
|  | Equivocal | 0.60 [0.59-0.61] | 0.59 [0.57-0.61] | 0.37 [0.34-0.39] | 0.52 [0.47-0.57] |
|  | Negative | 0.69 [0.68-0.70] | 0.69 [0.68-0.70] | 0.75 [0.73-0.78] | 0.77 [0.74-0.80] |
| **Average AUC** |  | 0.74 | 0.72 | 0.62 | 0.72 |
|  |  |  |  |  |  |
| **NPV** | Negative | 0.993 [0.992-0.993] | 0.993 [0.992-0.993] | 0.968 [0.943-0.981] | 0.998 [0.997-0.999] |
| **PPV** | Positive | 0.006 [0.005-0.007] | 0.005 [0.004-0.007] | 0.010 [0.006-0.017] | 0.001[0.001-0.002] |
|  |  |  |  |  |  |
| **Sensitivity** | Positive | 0.82 [0.78-0.86] | 0.73 [0.61-0.81] | 0.89 [0.67-0.97] | 1.00 [0.72-1.00] |
|  | Equivocal | 0.50 [0.49-0.52] | 0.50 [0.47-0.52] | 0.06 [0.04-0.08] | 0.15 [0.10-0.21] |
|  | Negative | 0.55 [0.54-0.55] | 0.55 [0.54-0.55] | 0.25 [0.24-0.28] | 0.39 [0.39-0.40] |
| **Macro Sensitivity** |  | 0.62 | 0.59 | 0.4 | 0.51 |
| **Weighted Sensitivity** |  | 0.55 | 0.55 | 0.22 | 0.39 |
|  |  |  |  |  |  |
| **Accuracy** |  | 0.545 [0.543-0.546] | 0.546 [0.543-0.549] | 0.22 [0.20-0.24] | 0.39 [0.38-0.40] |
| **Weighted Precision** |  | 0.98 | 0.98 | 0.80 | 0.98 |
| **Weighted F1 Score** |  | 0.7 | 0.7 | 0.34 | 0.56 |
| Model’s performance evaluation table for training cohort and three validation cohorts. All metric estimates are accompanied by 95% confidence intervals. Metrics are determined as follows:  AUCs: the measure of area under the receiver-operator characteristic curves reported for each PRISM class.  Negative Predictive Value (NPV): the ratio of true negatives (defined here PRISM negative predictions that are ground truth negative) over all ground truth negatives in the cohort: $\frac{TN}{TN+FN}$  Positive Predictive Value (PPV): the ratio of true positives over all predicted positives in the cohort: $\frac{TP}{TP+FP}$  Calculation   1. For UKBB: $\frac{53}{53 + 266+10048} =0.005$ 2. For HMDR: $\frac{16}{16+380+1158} = 0.01$ 3. For AoU:$\frac{10}{10+151+7358}= 0.001$   Sensitivity: the model’s true recall rate, defined as the ratio of true label to all ground truth labels for each PRISM class: ${Sensitivity}_{L} = \frac{TL}{TL + FL}, L = P, E, N$  Macro sensitivity: arithmetic mean of the sensitivity of each class: $\frac{1}{3}(\frac{TP}{TP+FP}+\frac{TE}{TE+FE}+\frac{TN}{TN+FN})$  Weighted sensitivity: the mean of each class’s sensitivity weighted by that class’s number of true sample size $=\frac{S_{P} \cdot{Sensitivity}_{P} + S_{E} \cdot{Sensitivity}_{E} + S_{N} \cdot{Sensitivity}_{N}}{N}$  Accuracy: Ratio of total correct predictions over all predictions =$\frac{TP + TE + TN}{(TP + FP) + (TE + FE) + (TN + FN)} =\frac{TP+TE+TN}{N}$  Weighted Precision: Precision is the ratio of true class calls over all predicted calls in that class  $\mathrm{Precision} = \frac{True Label}{All predicted label}$  Weighted by each class's true sample size (S_L_) and divided by the total N:  $Weighted Precision = \frac{S_{N} \cdot{Precision}_{N} + S_{E} \cdot{Precision}_{E} + S_{P} \cdot{Precision}_{P}}{N}$  Calculation   1. For UKB:   ${Precision}_{N}=\frac{49745}{49745+350+3}=0.99, {Precision}_{E}=\frac{603}{31231+603+17}=0.02, {Precision}_{P}= \frac{53}{10048+266+53}=0.005$  $S_{N}=91024,S_{E}=1219,S_{P}=73, N=92316$  $Weighted precision = \frac{(91024 * 0.99) +(1219*0.02)+(73*0.005)}{92316} =0.98$   2. For HMDR:   ${Precision}_{N}=\frac{457}{457+14+1}=0.97, {Precision}_{E}=\frac{23}{183+23+1}=0.1, {Precision}_{P}= \frac{16}{1158+380+16}=0.01$  $S_{N}=1798,S_{E}=417,S_{P}=18, N=2233$  $Weighted precision = \frac{(1798 * 0.97) +(417*0.1)+(18*0.01)}{2233} =0.80$   3. For AoU:   ${Precision}_{N}=\frac{5700}{5700+10+0}=0.998, {Precision}_{E}=\frac{28}{1417+28+0}=0.02, {Precision}_{P}= \frac{10}{7358+151+10}=0.001$  $S_{N}=14475,S_{E}=189,S_{P}=10, N=14674$  $Weighted precision = \frac{(14475* 0.99) +(189*0.02)+(10*0.001)}{14674} =0.98$  Weighted F1 score: the harmonic mean of recall and precision  Per-class F1 is the harmonic mean of that class's Precision and Sensitivity:  ${F1}_{L} = \frac{2 \cdot{Precision}_{L} \cdot{Sensitivity}_{L}}{{Precision}_{L} + {Sensitivity}_{L}}, L = P, E, N$  Weighted by each class's true sample size (S_L_) and divided by the total N:  $Weighted F1 = \frac{S_{P} \cdot{F1}_{P} + S_{E} \cdot{F1}_{E} + S_{N} \cdot{F1}_{N}}{N}$  Abbreviation:  P, E, N: the three PRISM classes, Positive, Equivocal, Negative  TP, TE, TN: True Positives, True Equivocals, True Negatives (correct predictions for each class)  FP, FE, FN: False Positives, False Equivocals, False Negatives (false predictions for each class)  S = Number of true instances in a class  N = total sample size across all classes (N = S_p_ + S_E_ + S_N_) | | | | | |

| **Table S6. Characteristics of individuals in each PRISM output class in the UKB test cohort and HMDR** | | | |
| --- | --- | --- | --- |
| **UKB test cohort** | | | |
|  | **Positive**  **(n=10367,11.2%)** | **Equivocal**  **(n=31851, 34.5%)** | **Negative**  **(n= 50098, 54.3%)** |
| Age median (Q1, Q3) | 62.4 (62.4, 66.4) | 64.4 (62.1, 66.9) | 51.8 (46.6, 56.4) |
| Sex (%) | 4407(42.5%) male, 5960(56.5%) female | 15552(48.9%) male, 16299(51.2%) female | 22355 (44.6%) male, 27743(55.4%) female |
| CH Status |  |  |  |
| CHIP (n = 5429) | 686 (6.6%) | 2658 (8.3%) | 2085 (4.2%) |
| CCUS (n = 458) | 266 (2.6%) | 73 (0.23%) | 119 (0.24%) |
| No CH (n=86429) | 9415 (90.8%) | 29120 (91.4%) | 47894 (95.6%) |
| (Non-clonal cytopenia, n =6175) | 2763 (26.7%) | 935 (2.9%) | 2477 (4.9%) |
| CHRS Risk Group |  |  |  |
| High-risk (n =42) | 42 (0.4%) | 0 (0%) | 0 (0%) |
| Intermediate-risk (n = 462) | 259 (2.5%) | 157 (0.5%) | 46 (0.1%) |
| Low-risk (n = 5383) | 651 (6.3%) | 2574 (8.1%) | 2158 (4.3%) |
| **HMDR cohort** | | | |
|  | **Positive**  **(n=1554, 69.6%)** | **Equivocal**  **(n=207, 9.3%)** | **Negative**  **(n= 472, 21.1%)** |
| Age, median (Q1, Q3) | 70.3 (61.1, 78.0) | 68.5 (64.3, 74.3) | 46.5 (36.4, 53.2) |
| Sex (%) | 709(45.6%) male, 845(54.4%) female | 99(47.8%) male, 108(52.2%) female | 291(61.7%) male, 181(38.3%) female |
| CH Status |  |  |  |
| CHIP (n = 206) | 105 (6.8%) | 63 (30.4%) | 38 (8.0%) |
| CCUS (n = 602) | 580 (37.3%) | 14 (6.8%) | 8 (1.7%) |
| No CH (n = 1425) | 869 (55.9%) | 130 (62.8%) | 426 (90.3%) |
| (Non-clonal cytopenia, n = 818)) | 652 (41.9%) | 27 (4.9%) | 139 (29.4%) |
| CHRS Risk Group |  |  |  |
| High-risk (n = 176) | 176 (11.3%) | 0 (0%) | 0 (0%) |
| Intermediate-risk (n = 373) | 350 (22.5%) | 13 (6.3%) | 10 (2.1%) |
| Low-risk (n = 259) | 159 (10.2%) | 64 (30.9%) | 36 (7.6%) |
| All categorical values are summarized as number and percentage, N (%). Continuous values are summarized as median (interquartile range). Data are summarized for the UK Biobank (UKB) test cohort and the hematologic malignancy data repository (HMDR) cohorts  Abbreviations: clonal hematopoiesis of intermediate potential (CHIP), clonal cytopenia of undetermined significance (CCUS), clonal hematopoiesis risk score (CHRS). | | | |

| **Table S7. Myeloid neoplasia (MN) outcomes by CHRS risk group assignment** | | | | |
| --- | --- | --- | --- | --- |
|  | UKB training cohort  (n = 369260) | UKB test cohort  (n =92316) | HMDR  (n = 2233) | All of Us  (n =14674) |
| CHRS risk Group |  |  |  |  |
| High-risk |  |  |  |  |
| No MN | 69 (46%) | 22 (52%) | 164 (93.2%) | 13 (76.5%) |
| Incident MN | 81 (54%) | 20 (48%) | 12 (6.8%) | 4 (23.5%) |
| Intermediate-risk |  |  |  |  |
| No MN | 1662 (90.8%) | 419 (90.7%) | 369 (98.9%) | 176 (96.2%) |
| Incident MN | 169 (9.2%) | 43 (9.3%) | 4 (1.1%) | 7 (3.8%) |
| Low-risk |  |  |  |  |
| No MN | 20834 (99.2%) | 5332 (99.05%) | 257 (99.2%) | 509 (98.6%) |
| Incident MN | 175 (0.8%) | 51 (0.95%) | 2 (0.8%) | 7 (1.4%) |
| No CH |  |  |  |  |
| No MN | 345378 (99.7%) | 86208 (99.7%) | 1423 (99.9%) | 13822 (99.0%) |
| Incident MN | 892 (0.3%) | 221 (0.3%) | 2 (0.1%) | 136 (1.0%) |
| For each cohort, the number and percentage, N (%), of individuals with incident MN and without incident MN (no MN) is shown according to CHRS risk group assignment.  Abbreviations: clonal hematopoiesis risk score (CHRS). Individuals without CH are labeled as “No CH”. | | | | |

| **Table S8. Characteristics of incident MN cases in each PRISM output class in the UKB test cohort** | | | |
| --- | --- | --- | --- |
|  | Positive (n=170) | Equivocal (n=94) | Negative (n=71) |
| Cytopenia | 53 (31.2%) | 5 (5.3%) | 4 (5.6%) |
| CH status |  |  |  |
| CHIP | 46 (27.1%) | 1 (1.1%) | 13 (18.3%) |
| CCUS | 26 (15.3%) | 28 (29.8%) | 0 (0%) |
| No CH | 98 (56.6%) | 65 (69.1%) | 58 (81.7%) |
| CHRS |  |  |  |
| Low-risk | 22 (12.9%) | 17 (18.1%) | 12 (16.9%) |
| Intermediate-risk | 30 (17.6%) | 12 (12.8%) | 1 (1.4%) |
| High-risk | 20 (11.8%) | 0 (0%) | 0 (0%) |
| Data are summarized for the UKB test cohort. All categorical values are summarized as number and percentage, N (%). Individuals without CH are labeled as “No CH”.  Abbreviations: myeloid neoplasm (MN); clonal hematopoiesis of intermediate potential (CHIP), clonal cytopenia of undetermined significance (CCUS), clonal hematopoiesis risk score (CHRS). | | | |

**Supplementary Figures:**

|  | Page # |
| --- | --- |
| List of Supplementary Figures |  |
| Figure S1: Consort diagram for the UKB training and test cohorts | 13 |
| Figure S2: Consort diagram for the HMDR cohort | 14 |
| Figure S3: Consort diagram for the All of Us cohort | 15 |
| Figure S4: Feature and Architecture Selection. | 16 |
| Figure S5: Platelet Distribution Width | 19 |
| Figure S6: Mutation Profiles of Cohorts | 20 |
| Figure S7: Confusion Matrices of Cohorts | 21 |
| Figure S8: PRISM benchmarking against CCRS | 22 |
| Figure S9: PRISM benchmarking against blood count and age | 23 |

**Figure S1. Consort diagram for the UKB training and test cohorts.** The UK Biobank is a long-term prospective registry of 502,490 individuals with ages ranging between 40 and 69 years at recruitment and followed over a decade. There were **461,576 participants eligible for study inclusion based on available sequencing and CBC data. Stratified splitting was used to divide the eligible population into training and test cohorts.**

**Figure S2. Consort diagram for the HMDR cohort**: The Hematologic Malignancies Data Repository (HMDR) is a registry of clinical, genetic and outcomes data of patients evaluated at Dana-Farber Cancer Institute in hematology and hematologic malignancies clinics. From a total of 4,132 individuals in the HMDR without hematologic malignancy diagnoses at the time of initial evaluation, **2233 individuals** had available CBC laboratory data and clinical sequencing data. This external clinical cohort was used as a “real-world” clinical dataset for model validation.

**Figure S3. Consort diagram for the All of Us cohort**: The All of Us Research Program population database is a United States based population cohort with integrated electronic health record, survey and whole genome sequencing (WGS) data collected from adults consented to study participation between May 31, 2017 to October 1, 2023. At the time of analysis, health data from >870,000 participants were available. CH variant calls derived from whole-genome sequencing were available for 276,542 individuals with linked laboratory measurements. Laboratory features included complete blood count parameters and related hematologic indices extracted from EHR data and we excluded individuals who lacked temporal alignment between sequencing and laboratory measurements as well as those who had implausible laboratory values indicative of clerical error with data entry. A final cohort of 14,674 remainder for external validation.

**Figure S4: Feature and Architecture Selection.** A. Initial list of putative feature inputs. B. Correlation heatmap of complete blood count measures displaying the Spearman correlation coefficient between each pair of complete blood count parameters. 3 pairs had correlations higher than 0.9 - hematocrit and hemoglobin, WBC and neutrophil, reticulocyte and high-scattering reticulocyte. Consequently, WBC, hematocrit and high-scattering reticulocyte were dropped from the input feature list. C. Model architectures evaluated included logistic regression (LogReg), K-nearest neighbors (KNN), decision tree (DT), Random Forest (RF), extreme gradient boosting (XGB), adaptive boosting (AdaB), gradient boosting (GradB), bagging bootstrap aggregation (Bagging), and light gradient boosting machine (LGBM). Performances were comparable for LogReg, RF, GradB, and LGBM classifiers. Random Forest architecture was chosen as the final model due to its handling of redundancies, ability to capture nonlinear effects, and its being computationally less intensive than LGBM. A Balanced Random Forest model was ultimately selected as final architecture to circumvent the class imbalance in our dataset. Each tree within the forest was trained on a randomly sampled balanced subset of the training data so that it trains on an equal representation of all classes. D. Boruta, a wrapper package around Random Forest that selects features that are most relevant to the dataset, was implemented to eliminate 5 features (sex, mean corpuscular hemoglobin concentration, basophil count, mean platelet volume, and nucleated red blood cell count) that were not contributing to model decision-making.

**(A)**

- Demographic parameters:
  - Age
  - Sex
- CBC parameters (23 available laboratory values):
  - White blood cell count (WBC), red cell blood count (RBC), hemoglobin concentration (Hbconc), hematocrit (haematocrit), mean corpuscular volume (MCV), mean corpuscular hemoglobin (MCHb), mean corpuscular hemoglobin concentration (MCHbconc), red cell distribution width (RDW), platelet count (platelet), platelet crit (plateletcrit – defined as the measure of percentage of total volume of blood occupied by platelets $\frac{platelet count ({10}^{3}/\mu L) \times mean platelet volume (MPV)(fL)}{10,000}$), mean platelet volume (MPV), platelet distribution width (PDW), lymphocyte count (lymphocyte), monocyte count (monocyte), neutrophil count (neutrophil), eosinophil count (eosinophil), basophil count (basophil), nucleated red blood cell count (nucRBC), reticulocyte count (reticulocyte), mean reticulocyte volume (MRV), mean sphered cell volume (MspCV), immature reticulocyte count (immret), high scattering reticulocyte count (hiscatret)

**(B)**

**(C)	(D)**

**Figure S5: Platelet Distribution Width & Giant Platelet Threshold.** (left) Violin plot of platelet distribution width (PDW) with the central vertical line with caps highlighting the range of PDW. Q1, Q2 and Q3 are marked on the plot. (right) Histogram displays the distribution of PDW; the vertical blue line marks the third quartile (Q3), which defines the selected threshold for marking the presence of giant platelets.

**Figure S6: Mutation profiles of cohorts.** (left) Histogram of 15 most mutated genes plotted for subpopulations with and without MN incidence for (A) UKB training cohort, (B) UKB test cohort, (C) HMDR cohort, (D) All of Us cohort. (right) Fraction of individuals with 1, 2 or 3 mutated genes in subpopulations with and without MN incidence for (A) UKB training cohort, (B) UKB test cohort, (C) HMDR cohort, (D) All of Us cohort.

**Figure S7: Confusion matrices of cohorts.** PRISM classification performance evaluated by comparing the model’s predicted class assignments against ground-truth labels for (A) UKB test cohort, (B) HMDR cohort, (C) All of Us cohort. Rows represent true class labels, and columns represent the predicted classes by PRISM. Diagonal entries indicate correct classification while off-diagonal elements indicate misclassifications.

**Figure S8: PRISM benchmarking against CCRS.** (A) 3x3 table showing the correlation between CHRS risk groups and CCRS risk groups. (B) 2 x 3 table showing how CCRS risk groups correlate with m-CH scores. (C) 2 x 3 table showing how CHRS risk groups correlate with m-CH scores. (D) Distribution of PRISM class assignment for each CCRS risk group where PRISM classes are indicated as follows: positive (green), negative (blue), and equivocal (red). CCRS risk groups are abbreviated as HR for high-risk, IR for intermediate-risk, and LR for low-risk. Bars are annotated with the N (%) for each PRISM class. Data are shown for the subset of UK Biobank participants with cytopenias in the test cohort.

**Figure S9: PRISM benchmarking against blood counts and age.** PRISM positive class performance in capturing high-risk CH individuals compared against thresholds of MCV, RDW, age and presence of cytopenia. (A) Table listing the variables benchmarked, their threshold values (where 1 = positive for categorical values), number and fraction of individuals above the threshold who will be screened, number and fraction of high-risk CHRS individuals who fall above the thresholds, and number needed to screen (NNS) – number of individuals to be screened to detect one true high-risk CHRS plotted for each criterion. (B) Sensitivity or the fraction of high-risk CHRS detected plotted against the percentage of population screened for each criterion. Although thresholds of RDW and MCV screen a lower fraction of individuals (lower NNS than the PRISM positive class), using these features at defined cutoffs has a lower sensitivity for high-risk CH detection.

Example PRISM use cases randomly selected from the HMDR clinical cohort.

PRISM web calculator:

<https://chideaslab.shinyapps.io/chmnapp/>
